# High Expression of THY1 in Intestinal Gastric Cancer as a Key Factor in Tumor Biology: A Poor Prognosis-Independent Marker Related to the Epithelial–Mesenchymal Transition Profile

**DOI:** 10.3390/genes15010028

**Published:** 2023-12-24

**Authors:** Paulo Rohan, Everton Cruz dos Santos, Eliana Abdelhay, Renata Binato

**Affiliations:** Stem Cell Laboratory, Division of Specialized Laboratories, Instituto Nacional de Câncer (INCA), Rio de Janeiro 20230-130, RJ, Brazil; rohanphn@gmail.com (P.R.); evertoncruzsantos@gmail.com (E.C.d.S.); eabdelhay@inca.gov.br (E.A.)

**Keywords:** gastric cancer, intestinal-type gastric cancer, THY1, molecular profile

## Abstract

Gastric cancer (GC) is an important cancer-related death worldwide. Among its histological subtypes, intestinal gastric cancer (IGC) is the most common. A previous work showed that increased expression of the *THY1* gene was associated with poor overall survival in IGC. Furthermore, it was shown that IGC tumor cells with high expression of *THY1* have a greater capacity for tumorigenesis and metastasis in vitro. This study aimed to identify molecular differences between IGC with high and low expression of *THY1*. Using a feature selection method, a group of 35 genes were found to be the most informative gene set for *THY1*^high^ IGC tumors. Through a classification model, these genes differentiate *THY1*^high^ from *THY1*^low^ tumors with 100% of accuracy both in the test subset and the independent test set. Additionally, this group of 35 genes correctly clustered 100% of the samples. An extensive validation of this potential molecular signature in multiple cohorts successfully segregated between *THY1*^high^ and *THY1*^low^ IGC tumors (>95%), proving to be independent of the gene expression quantification methodology. These genes are involved in central processes to tumor biology, such as the epithelial–mesenchymal transition (EMT) and remodeling of the tumor tissue composition. Moreover, patients with *THY1*^high^ IGC demonstrated poor survival and a more advanced clinicopathological staging. Our findings revealed a molecular signature for IGC with high *THY1* expression. This signature showed EMT and remodeling of the tumor tissue composition potentially related to the biology of IGC. Altogether, our results indicate that *THY1*^high^ IGC tumors are a particular subset of tumors with a specific molecular and prognosis profile.

## 1. Introduction

Gastric cancer (GC) is a complex and aggressive disease that currently ranks fifth in incidence and fourth as the leading cause of cancer-related death worldwide [1]. Most cases of GC are diagnosed late, mainly due to their nonspecific symptomatology, contributing to the severity of the disease at diagnosis [2,3]. Thus, despite several advances regarding anticancer therapy in recent years, the prognosis of GC remains unsatisfactory, with a 5-year survival rate of approximately 20%, high recurrence rates, and distal metastasis events [4]. At the histological level, adenocarcinoma is the most common type of GC, accounting for 95% of all cases [5,6], and is divided into two subtypes according to Lauren’s histological classification, intestinal GC (IGC) and diffuse GC (DGC), with different etiological, epidemiological, and genetic characteristics [7]. Among these subtypes, IGC is the most common, accounting for 70% of cases [8,9]. This subtype is markedly associated with the multistep progression that can be initiated by chronic *Helicobacter pylori* infection, as proposed by the model of human gastric carcinogenesis [10,11,12]. Due to its complexity and biological heterogeneity, several studies have focused on biomarkers to understand the development and progression of IGC. In this sense, studies have demonstrated that the *THY1* gene is a potential marker of poor prognosis for patients with GC, including one from our group that demonstrated this specifically in IGC patients [13,14,15,16].

Thy-1, or CD90, is a glycosylphosphatidylinositol (GPI)-anchored cell surface protein that lacks a transmembrane domain [17,18]. This protein has a group of ligands composed of different integrins, including the integrins αvβ3, αvβ5, α5β1, αMβ2, and αCβ2 [19,20,21,22,23]. In a physiological context, Thy-1 is mainly expressed in neurons, thymocytes, fibroblasts, and mesenchymal stem cells, where it is related to functions such as cell adhesion, differentiation, migration, and proliferation [24,25,26,27]. The high expression of Thy-1 has been associated with several types of cancer; however, its impact on the prognosis of the disease appears to be context-dependent. In the context of hepatocellular and renal carcinomas, high expression of the *THY1* gene has already been associated with poor prognosis and as a marker of cancer stem cells with tumorigenic and metastatic capacity [28,29]. In contrast, in ovarian cancer and nasopharyngeal carcinoma, high expression of *THY1* was associated with tumor suppression and was involved in the suppression of metastasis [30,31]. These characteristics highlight that the role of the *THY1* gene in prognosis depends on the pathological context. In the context of GC, together with studies that demonstrate *THY1* as a potential marker of poor prognosis, the level of *THY1* expression in tumor cells is associated with a greater capacity for tumorigenesis, proliferation, adhesion, and metastasis, emphasizing its importance in the context of the origin and progression of the disease [32,33,34,35]. Nonetheless, the molecular mechanisms underlying the tumor cell behavior and poor prognosis observed in patients with IGC tumors expressing high levels of *THY1* have not yet been clarified, with few studies addressing this issue [36]. Therefore, these key questions must be addressed to understand the molecular mechanisms implicated in the biology of these tumors.

To address this knowledge gap, we performed bioinformatics analyses using machine learning models to investigate distinct molecular patterns between IGC tumors with high and low expression of the *THY1* gene. We systematically addressed this issue by using expression data from different cohorts with different transcriptomic methodologies and patients from different geographic backgrounds. Through this approach, we identified a robust molecular signature of 35 genes for *THY1*^high^ IGC tumors. This molecular signature reveals key processes in the biology of *THY1*^high^ IGC tumors, such as the epithelial–mesenchymal transition and processes involved in the remodeling of the tumor tissue composition and cellular interactions. Moreover, we showed that patients with *THY1*^high^ IGC tumors have poor prognosis in different aspects.

Altogether, the findings provide knowledge regarding the biology of IGC tumors with high expression of *THY1*, which can help us to understand not only its biological behavior and molecular background but also the worse prognosis of *THY1*^high^ patients.

## 2. Materials and Methods

### 2.1. Data Acquisition and Preprocessing

**Figure 1:**
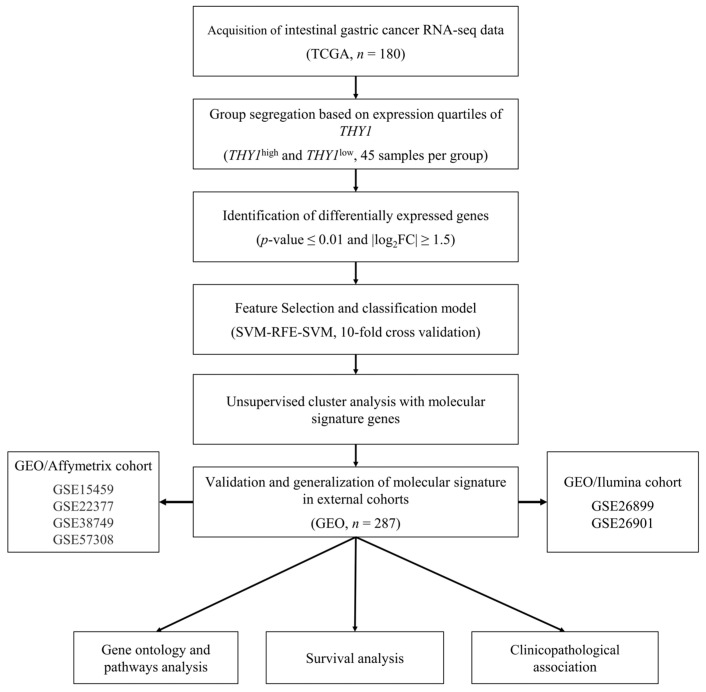
Workflow of the study. RNA-seq and clinical data for IGC patients (*n* = 180) were obtained from the TCGA database. These samples were then segregated according to the level of *THY1* gene expression, with the upper quartile representing the *THY1*^high^ group (*n* = 45) and the lower quartile representing the *THY1*^low^ group (*n* = 45). Differential gene expression analysis was performed to obtain DEGs, with the *THY1*^low^ group used as a reference for comparison. Genes that passed the cutoff criteria of *p*-value ≤ 0.01 and |log2FC| ≥ 1.5 were considered DEGs. The total group of 90 samples was divided into a training set (*n* = 63) and a test set (*n* = 27), and the expression level of the DEGs was used as input to an algorithm of recursive feature elimination with the support vector machine model. The resulting gene group was used to construct a classification support vector machine model. After that, the gene group was used in unsupervised cluster analysis. Clinical and microarray gene expression data for IGC patients (*n* = 287) were obtained from a total of six studies through the GEO database for validation of the group of genes. Finally, Gene Ontology and pathway analyses were performed for the molecular signature. Survival analyses and clinicopathological association were executed using the different cohorts.

RNA-seq data of 180 patients with IGC, as well the clinical data related to them, were obtained from The Cancer Genome Atlas (TCGA) Project through the Genomic Data Commons (GDC) portal using the TCGAbiolinks package (version 2.25; TCGAbiolinks, RRID:SCR_017683) from R/Bioconductor (version 3.17 (http://www.bioconductor.org/, accessed on 5 May 2023) Bioconductor, RRID:SCR_006442) in R software (version 4.2.2 (https://www.r-project.org/, accessed on 3 March 2023); R software, RRID:SCR_001905) [37]. The design and workflow of this study are shown in Figure 1. Additionally, two small independent sample sets (GSE191275, *n* = 10 and GSE193453 *n* = 4) were obtained using the GEOquery package (version 2.38.4; GEOquery, RRID:SCR_000146) and processed in the same way as TCGA data.

### 2.2. Segregation of Samples According to the Expression Level of THY1

After obtaining the gene expression matrix, it was used as input to the package DESeq2 (version 1.38.3; DESeq2, RRID:SCR_015687), with which data normalization was performed using the median of ratios (MRM) method [38]. After normalization, *THY1* gene expression quartiles were calculated: the *THY1*^low^ group was established as the lower quartile of expression, and the *THY1*^high^ group was established as the upper quartile, with 45 IGC patients per group, totaling 90 samples included in this study. Principal component analysis (PCA) was applied to visualize segregation between the groups.

### 2.3. Differential Gene Expression Analysis and Differentially Expressed Gene Identification

Differentially expressed genes (DEGs) were identified using the DESeq2 package [38]. For this, comparison between the *THY1*^low^ and *THY1*^high^ groups (45 samples in each group) was performed. The *THY1*^low^ group was considered the reference group, which means that the identified DEGs were upregulated or downregulated in the *THY1*^high^ group. After the analysis, the biomaRt Package (version 2.54.1; biomaRt, RRID:SCR_019214) was used to filter only protein-coding genes, totaling 18,907 final genes [39]. For DEG identification, an adjusted *p*-value ≤ 0.01 and |log2FC| ≥ 1.5 were considered statistically significant. The package EnhancedVolcano (version 1.18.0; EnhancedVolcano, RRID:SCR_018931) was used to visualize the distribution of DEGs.

### 2.4. Feature Selection and Construction of a Supervised Machine Learning Model

To verify the existence of a genetic signature that can correctly discriminate between the *THY1*^low^ and *THY1*^high^ groups, we first selected the most informative genes through the recursive feature elimination with support vector machine (SVM-RFE) algorithm. We chose this algorithm based on its demonstrated performance in the context of cancer and gene selection [40]. For that, we first randomly split the 90 samples into a training set composed of 70% (63/90) and a test set composed of 30% (27/90). In the training set, 32 and 31 samples were from the *THY1*^high^ and the *THY1*^low^ group, respectively. In the test set, 13 samples were from *THY1*^high^ and 14 were from *THY1*^low^. Then, the training set was used to perform SVM-RFE with 10-fold cross-validation through the gene expression levels transformed by the variance stabilizing transformation (VST) method and scaled by z-score normalization as input [38]. Then, the final support vector machine (SVM) model was constructed using only the gene expression levels of genes selected by SVM-RFE and performance was evaluated during construction using average accuracy with 10-fold cross-validation. The hyper-parameters’ tuning was carried out through GridSearchCV, and the linear kernel was chosen [41]. After building the model, it was tested through the Confusion Matrix and area under the ROC curve (AUC) using the TCGA test set (*n* = 27) and the two small independent sample sets (GSE191275 and GSE193453, *n* = 7), which were processed in the same way as the TCGA cohort [42,43]. All the above steps were implemented using the scikit-learn package (version 1.3.2; scikit-learn, RRID:SCR_002577) in the Python programming language (version 3.11; Python programming language, RRID:SCR_008394) [44,45].

### 2.5. Unsupervised Cluster Analysis

By using expression data of genes in the molecular signature, normalized, and transformed by the VST method and scaled to Z-score, hierarchical clustering analysis was performed using the ComplexHeatmap package (version 2.14.0; ComplexHeatmap, RRID:SCR_017270) [38,46]. Regarding the grouping of samples, the similarity metric used was the Euclidean distance, and the grouping method was Ward’s linkage. For the k-means clustering, the same data were used as input. Then, we used the scikit-learn package (version 1.3.2; scikit-learn, RRID:SCR_002577) and specified the number of clusters as two.

### 2.6. Molecular Signature Validation

To verify whether the identified molecular signature is capable of separating IGC samples from other populations and methodological platforms, we performed unsupervised cluster analysis with Gene Expression Omnibus (GEO) data using the molecular signature genes. The evaluated studies were GSE15459 (99 IGC samples), GSE22377 (24 IGC samples), GSE26899 (59 IGC samples), GSE26901 (82 IGC samples), GSE38749 (4 IGC samples), and GSE57308 (19 IGC samples), totaling 287 samples. Of these, two studies used the chip Illumina HumanHT-12 V3.0 expression beadchip (GSE26899 and GSE26901, *n* = 141) and four studies used the chip Affymetrix Human Genome U133 Plus 2.0 Array (GSE15459, GSE22377, GSE38749, and GSE57308, *n* = 146). Then, the data were grouped into two main cohorts, the GEO/Illumina (GSE26899 and GSE26901, *n* = 141) and GEO/Affymetrix (GSE15459, GSE22377, GSE38749, and GSE57308, *n* = 146) cohorts. The data were obtained using the GEOquery package (version 2.38.4; GEOquery, RRID:SCR_000146), and expression data were processed with the limma package (version 3.56.2; limma, RRID:SCR_010943) using the quantile normalization for the GEO/Illumina and the robust multichip average (RMA) normalization method for the GEO/Affymetrix cohort [47,48]. After obtaining gene expression data, the samples were divided into *THY1*^low^ and *THY1*^high^ groups based on quartiles of expression, with 35 patients in the *THY1*^high^ group (upper quartile) and 36 in the *THY1*^low^ group (lower quartile) for the GEO/Illumina cohort, and 37 patients in the *THY1*^high^ and 37 in the *THY1*^low^ group for the GEO/Affymetrix cohort. A total of 145 samples were included in the analysis.

### 2.7. Gene Ontology and Pathway Analysis

Molecular signature genes were used for overrepresentation analysis (ORA) through the clusterProfiler package (version 4.10.0; clusterProfiler, RRID:SCR_016884) [49,50]. For this analysis, the significance limit was configured for *p*-value ≤ 0.05. The gene sets used for the analysis were obtained through the Molecular Signature Database (MSigDB, version 3.0; MSigDB, RRID:SCR_016863) [51]. Within this database, gene sets consisting of the Gene Ontology Biological Process (GOBP), well-defined biological processes and states (Hallmarks), Kyoto Encyclopedia of Genes and Genomes (KEGG), WikiPathways, and Reactome were selected [52,53,54,55,56].

### 2.8. Survival Analysis and Clinicopathological Characteristics Association

After previous analyses, survival and clinicopathological information from the TCGA, GEO/Illumina, and GEO/Affymetrix cohorts were used for the remaining analyses. For the association between clinicopathological characteristics and the *THY1* expression groups, Fisher’s exact test was applied, and *p*-values ≤ 0.05 were considered statistically significant. Then, overall survival and recurrence-free survival analyses were performed between the *THY1*^low^ and *THY1*^high^ groups using the survival package (version 2.11-4; survival, RRID:SCR_021137). The Kaplan-Meier method was applied, and log-rank test *p*-values ≤ 0.05 were considered statistically significant.

### 2.9. Statistical Analysis

All statistical analyses were performed using R software (version 4.3.1). *p*-values ≤ 0.05 were considered statistically significant. When appropriate, correction for multiple tests was applied to generate an adjusted *p*-value using the Benjamini-Hochberg method for correction, and an adjusted *p*-value ≤ 0.05 was considered to be statistically significant [57].

## 3. Results

### 3.1. Segregation of THY1^high^ and THY1^low^ Groups

To identify molecular differences between intestinal gastric cancer (IGC) tumors with high and low expression of *THY1*, we collected data for 180 IGC patients with available RNA-seq data in the TCGA database. After data preprocessing and normalization by the median of ratios method MRM, these patients were separated according to *THY1* gene expression quartiles, with 45 patients considered to have high *THY1* gene expression (*THY1*^high^ group, referring to the upper quartile) and 45 considered to have low expression (*THY1*^low^ group, referring to the lower quartile). All subsequent analyses were based on this segregation, and the study workflow is summarized in Figure 1.

**Figure 2:**
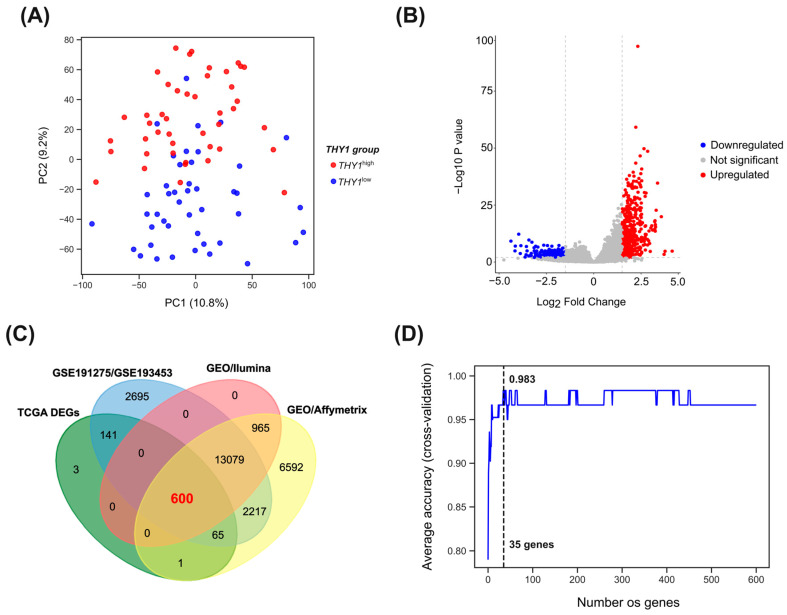
Gene expression pattern and feature selection between the *THY1*^high^ and *THY1*^low^ groups. (**A**) This figure shows a scatter plot of the principal component analysis. The *x*-axis represents principal component 1 (PC1), and the *y*-axis represents principal component 2 (PC2). The proportion described on the axes refers to the proportion of variance that these components explain. (**B**) This volcano plot depicts the DEGs identified in the analysis. The *x*-axis represents the log2-fold change, and the *y*-axis represents the adjusted *p*-value in −log10 values. The red color represents the upregulated genes in the *THY1*^high^ compared to the *THY1*^low^ group, considered as those who passed the cutoff point of *p*-value ≤ 0.01 and log2FC ≥ 1.5. The blue color represents the downregulated genes in the *THY*1^high^ compared to the *THY1*^low^ group, considered as those who passed the cutoff point of *p*-value ≤ 0.01 and log2FC ≤ −1.5. The gray color represents the genes that were not significant in our cutoff point. (**C**) Venn diagram showing overlap between genes that were measured on all transcriptomic platforms of the cohorts included in this study. (**D**) Line plot showing the average accuracy for all possibilities between 1 and 600 genes in the SVM-RFE algorithm.

The general characteristics of the patients can be found in Table S1. For a broad overview of this segregation, we performed principal component analysis (Figure 2A) using all the gene expression data, which showed that these two groups have distinct global expression patterns that can separate them.

### 3.2. Differential Gene Expression between THY1^high^ and THY1^low^ Groups

To identify differential gene expression between the *THY1*^high^ and *THY1*^low^ groups, we first excluded low counts and used only protein-coding genes, totaling 18,907 genes for this analysis. Furthermore, we used the *THY1*^low^ group as a reference for comparison, which means that the DEGs identified were upregulated or downregulated in the *THY1*^high^ group. As a result, 811 DEGs were identified, including 473 upregulated (*p*-value ≤ 0.01, Log2FC ≥ 1.5) and 338 downregulated (*p*-value ≤ 0.01, Log2FC ≤ −1.5) DEGs (Figure 2B). A list of all identified DEGs is provided in Table S2.

### 3.3. Feature Selection and Supervised Machine Learning Approach Reveals a Group of Genes with High Discriminatory Power for THY1^high^ IGC Tumors

To further reduce the DEGs obtained in the TCGA cohort for the most informative genes, we first eliminated the *THY1* gene from this set so as not to influence the classification function. Then, we kept only the genes that were present on all transcriptomic platforms of the cohorts included in this study (Affymetrix Human Genome U133 Plus 2.0 Array and Illumina HumanHT-12 V3.0 expression beadchip), with the aim of proceeding only with genes available on all platforms, totaling 600 genes (Figure 2C). We then applied an algorithm of SVM-RFE using gene expression levels transformed by the VST method and scaled by z-score normalization. For that, we first randomly split the 90 samples into a training set composed of 70% (63/90) and a test set composed of 30% (27/90). In the training set, 32 and 31 samples were from the *THY1*^high^ and the *THY1*^low^ groups, respectively. In the test set, 13 samples were from *THY1*^high^ and 14 from *THY1*^low^. Then, the training set was used for SVM-RFE with 10-fold cross-validation. As shown in Figure 2D, the highest average accuracy value with the lowest number of genes was found for a group of 35 genes (98.3% average accuracy).

**Figure 3:**
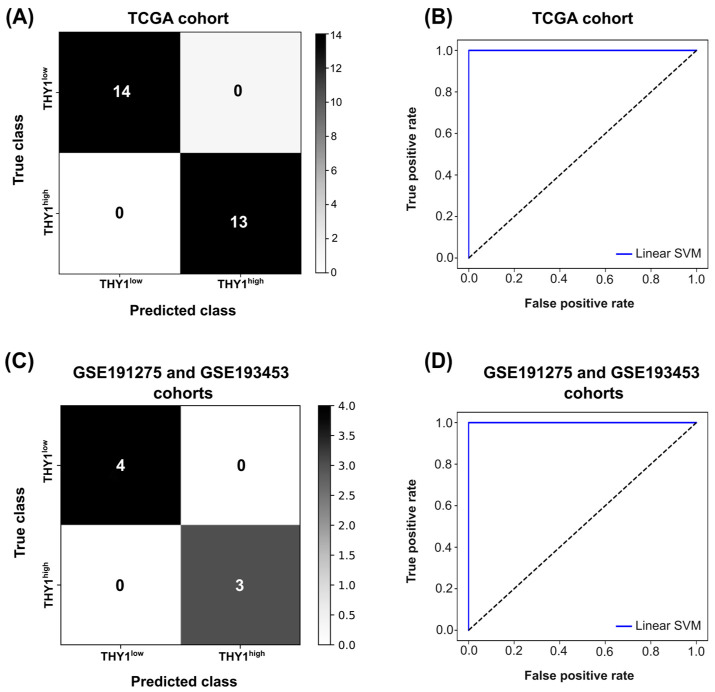
Evaluation of the classification performance of the 35 molecular signature genes in an SVM model. (**A**) Confusion matrix of the test set of the TCGA cohort (*n* = 27). The color gradient on the right represents the number of samples in each quadrant. (**B**) ROC-AUC curve of the test set of the TCGA cohort. The *x*-axis represents the false positive rate and the *y*-axis the true positive rate. The blue line represents the AUC for the linear SVM model. (**C**) Confusion matrix of the small sets of independent samples (GSE191275 and GSE193453 *n* = 7). The color gradient on the right represents the number of samples in each quadrant. (**D**) ROC-AUC curve of the small sets of independent samples (GSE191275 and GSE193453 *n* = 7). The *x*-axis represents the false positive rate and the *y*-axis the true positive rate. The blue line represents the AUC for the linear SVM model.

We then used this set of 35 genes to build the final SVM classification model. After training and testing the model, we found an overall accuracy of 100% (Figure 3A) and an AUC of 1.0 (Figure 3B), with all samples being correctly classified. To further address this predictive potential, we used RNA-seq data from two small sets of independent samples (GSE191275 *n* = 10 and GSE193453 *n* = 4) that were processed in the same way as the test group. After segregating the samples according to the *THY1* gene expression quartiles (*n* = 7), these were used as input for the classification model using the 35 genes. As a result, an accuracy of 100% (Figure 3C) and an AUC of 1.0 (Figure 3D) were achieved. These results highlight the high predictive capacity of this set of 35 genes and, therefore, we consider these genes as a molecular signature for *THY1*^high^ IGC tumors (Table 1).

### 3.4. Candidate Genes in the Molecular Signature Can Successfully Segregate between THY1^high^ and THY1^low^ IGC Tumors in an Unsupervised Manner

**Figure 4:**
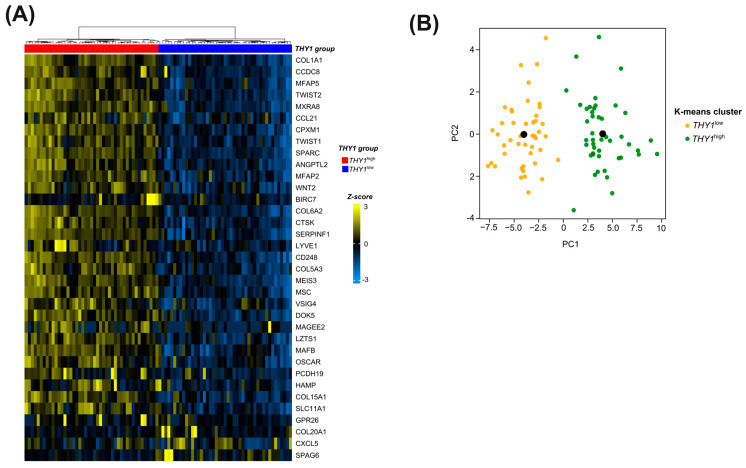
Unsupervised cluster analysis of *THY1* expression groups according to the expression levels of the 35 genes from the molecular signature in the TCGA cohort. (**A**) Heatmap of hierarchical clustering in the TCGA cohort. The lines represent each of the 35 molecular signature genes, and the columns represent each of the 90 samples. In the upper part of the figure, the dendrograms represent the hierarchical grouping of the samples based on the Euclidean distance between them using the Ward’s linkage grouping method. The first rectangle below the dendrogram represents the classification of cases, with the red one referring to the *THY1*^high^ group and the blue one to the *THY1*^low^ group. The Z score, which represents the range of colors used, was used to scale the expression data. (**B**) Two principal components of a principal component analysis representing the k-means clustering in the TCGA cohorts (*n* = 90). The black dot represents the cluster centroid.

To verify the capacity of the 35 genes to function as a molecular signature for *THY1*^high^ IGC tumors in a way that does not require prior model training and that can be applied independently of the transcriptomic methodology, we conducted unsupervised cluster analysis using the expression levels of only these genes. For this, we used the Euclidean distance as a similarity metric for the samples with the Ward’s linkage clustering method. As a result, correct clustering occurred in 100% of the samples. The heatmap in Figure 4A illustrates this distribution.

To further understand this group structure based on the 35 genes, we performed a k-means clustering analysis to validate the previously observed clustering. As a result, all samples grouped correctly (Figure 4B), reinforcing the previous results. It is interesting to note that these 35 genes were able to segregate between the two groups in a satisfactory manner in all methodologies applied, which strengthens the use of these genes as a molecular signature for these groups.

### 3.5. External Cohort Validation Reveals the Robustness and Generalization Capacity of the 35-Gene Molecular Signature for THY1^high^ IGC Tumors

To ensure that the results observed were not influenced by bias in the TCGA population or RNA-seq methodology, we used several external cohorts for validation of this molecular signature. We obtained data from six different studies in the GEO database (GSE15459, GSE22377, GSE26899, GSE26901, GSE38749, and GSE57308), covering patients of different nationalities with gene expression data obtained from different microarray platforms (Illumina HumanHT-12 V3.0 expression beadchip and Affymetrix Human Genome U133 Plus 2.0 Array), with a total of 287 IGC patients (Table S3). These studies were divided into two major cohorts, the GEO/Illumina cohort (GSE26899 and GSE26901, *n* = 141) and the GEO/Affymetrix cohort (GSE15459, GSE22377, GSE38749, and GSE57308, *n* = 146). Preprocessing and normalization of the data were carried out by the quantile normalization for the GEO/Illumina cohort and RMA for the GEO/Affymetrix cohort. We then separated the *THY1*^high^ and *THY1*^low^ groups based on *THY1* gene expression quartiles, with 35 patients in the *THY1*^high^ group (upper quartile) and 36 in the *THY1*^low^ group (lower quartile) for the GEO/Illumina cohort and 37 patients in the *THY1*^high^ and 37 in the *THY1*^low^ group for the GEO/Affymetrix cohort.

**Figure 5:**
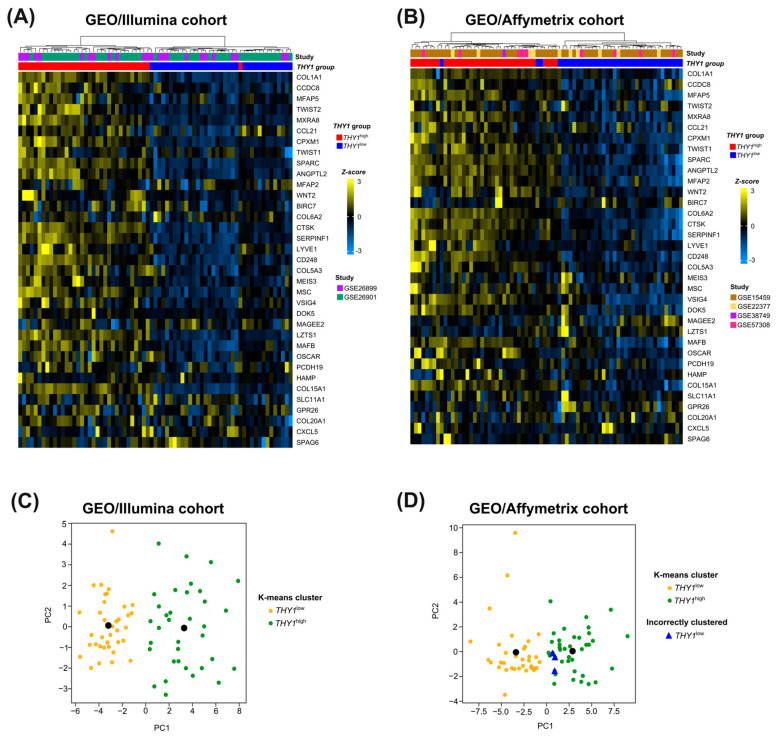
Unsupervised cluster analysis of *THY1* expression groups according to the expression level of the 35 genes of common molecular signature in the GEO cohorts. (**A**,**B**) Heatmap of hierarchical clustering in the GEO/Illumina (*n* = 71) and GEO/Affymetrix (*n* = 74) cohorts, respectively. The lines represent each of the 35 molecular signature genes, and the columns represent each sample. In the upper part of the figure, the dendrograms represent the hierarchical grouping of the samples based on the Euclidean distance between them using the Ward’s linkage grouping method. The first rectangle below the dendrogram represents the GEO studies. The second rectangle below the dendrogram represents the classification of cases, with the red one referring to the *THY1*^high^ group and the blue one to the *THY1*^low^ group. The Z-score, which represents the range of colors used, was used to scale the expression data. (**C**,**D**) Two principal components of a principal component analysis representing the k-means clustering in the GEO/Illumina (*n* = 71) and GEO/Affymetrix (*n* = 74) cohorts, respectively. The black dot represents the cluster centroid. The blue triangle represents samples from the *THY1*^low^ group that were clustered incorrectly.

Then, we applied the same previously unsupervised cluster analysis to these expression data. As shown in Figure 5A, a correct clustering of 98.6% (70/71) was achieved in the GEO/Illumina cohort, with one sample from the *THY1*^high^ group clustered with the wrong group. Furthermore, for the GEO/Affymetrix cohort, a correct clustering of 95.9% (71/74) was achieved, with three samples from the *THY1*^low^ group clustered with the wrong group (Figure 5B). Moreover, we applied the k-means clustering to additionally verify the distribution of these groups. Interestingly, 100% of the samples from the GEO/Illumina cohort were clustered correctly (Figure 5C), whereas for the GEO/Affymetrix cohort, the same correct clustering rate was maintained (95.9%), with three samples from the *THY1*^low^ group clustered incorrectly (Figure 5D).

Taken together, these results show that the molecular signature was able to successfully separate these groups. This demonstrates the discriminatory nature of this set of genes in a manner that is independent of the methodology used to obtain the gene expression data or geographic origin of the patients. Hence, these 35 genes can be considered a robust molecular signature and a consistent biological pattern for *THY1*^high^ IGC tumors.

### 3.6. Molecular Signature Genes of THY1^high^ IGC Tumors Are Involved in Key Processes of the Epithelial–Mesenchymal Transition and Remodeling of Tumor Tissue Composition

To understand the biological consequences of the genes involved in the identified molecular signature and provide context for such molecular changes, we performed ORA to identify the biological processes in which these genes are involved. To this end, we used as a reference a collection of gene sets from GOBP, Hallmarks, KEGG, WikiPathways, and Reactome databases.

As a result, several biological processes were found to be statistically significant (*p* ≤ 0.05). Among these, processes involved in remodeling of the tumor tissue composition (extracellular matrix organization, collagen formation, and regulation of angiogenesis), epithelial–mesenchymal transition (epithelial–mesenchymal transition, epithelial–mesenchymal transition in colorectal cancer, and positive regulation of epithelial–mesenchymal transition), and gain of aggressiveness characteristics (positive regulation of cell migration, positive regulation of cell motility, positive regulation of locomotion, and embryo development) were found enriched in the molecular signature.

The statistics of these processes and the list of genes involved in each process are summarized in Table 2. Altogether, these results indicate that the processes involved in the epithelial–mesenchymal transition (EMT) and processes that encompass different aspects of remodeling of the tumor tissue composition and a cell’s interaction within the tissue context are central processes for the biology of *THY1*^high^ IGC tumors.

### 3.7. THY1^high^ IGC Tumors Lead to Poor Survival with a Heterogeneous Clinicopathological Staging

**Figure 6:**
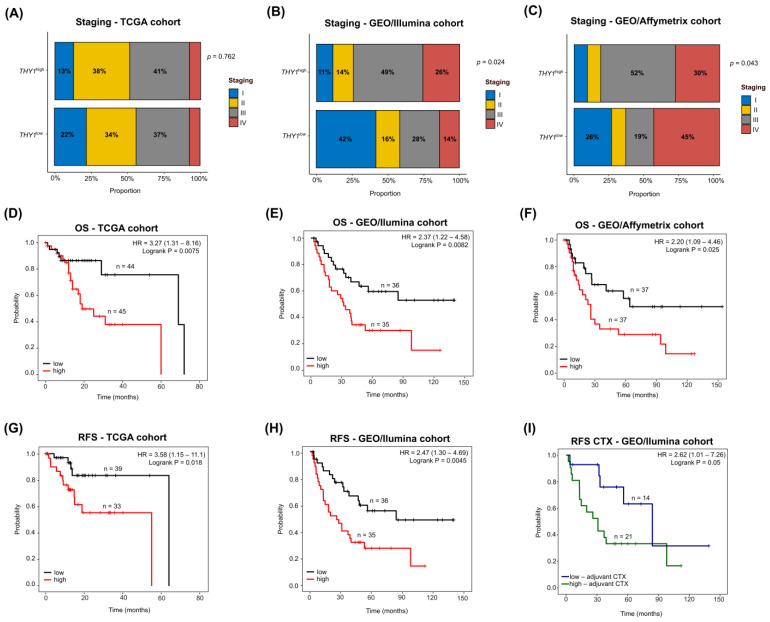
Clinicopathological association and survival analysis between the *THY1*^high^ and *THY1*^low^ groups. (**A**–**C**) Association between clinicopathological staging and the *THY1* group in the TCGA, GEO/Illumina, and GEO/Affymetrix cohorts, respectively. The association was tested using Fisher’s exact test. (**D**–**F**) Overall survival analysis between the *THY1*^high^ and *THY1*^low^ groups in the TCGA, GEO/Illumina, and GEO/Affymetrix cohorts, respectively. The red line refers to the *THY1*^high^ group, and the black line refers to the *THY1*^low^ group. (**G**,**H**) Recurrence-free survival analysis between the *THY1*^high^ and *THY1*^low^ groups in the TCGA and GEO/Illumina cohorts, respectively. The red line refers to the *THY1*^high^ group, and the black line refers to the *THY1*^low^ group. (**I**) Recurrence-free survival analysis between the *THY1*^high^ and *THY1*^low^ groups that had received the adjuvant chemotherapy regimen in the GEO/Illumina cohort. The green line refers to the *THY1*^high^ group, and the blue line refers to the *THY1*^low^ group. The *x*-axis represents the survival time in months, and the *y*-axis represents the probability of survival.

To verify the prognostic impact of high *THY1* gene expression in patients with IGC, we first tested the association between the established groups (*THY1*^high^ vs. *THY1*^low^) and clinicopathological staging of the patients. As a result, no significant association was observed between the *THY1* groups and the clinicopathological staging of patients in the TCGA cohort (Figure 6A). However, in both GEO/Illumina and GEO/Affymetrix cohorts, the *THY1*^high^ group demonstrated a significant association with more advanced stages of the disease. As shown in Figure 6B, in the GEO/Illumina cohort, 75% of *THY1*^high^ cases were found in advanced stages (III–IV), compared to 42% of *THY1*^low^ cases (*p* = 0.024). Furthermore, the same pattern was observed in the GEO/Affymetrix cohort (Figure 6C), with 82% of THY1^high^ cases found in advanced stages (III–IV) compared to 64% of *THY1*^low^ cases (*p* = 0.043). These results indicate that although a certain heterogeneity is observed between the cohorts, both groups tend to be distributed in all stages, but with the *THY1*^high^ group preferentially in advanced stages.

Then, we performed survival analysis to verify the impact of *THY1* groups in patient survival. Regarding overall survival (OS), patients with *THY1*^high^ IGC tumors consistently had poor OS across all three cohorts. In the TCGA cohort (Figure 6D), patients with *THY1*^high^ IGC tumors had poorer OS (log-rank *p* = 0.0075, hazard ratio = 3.27, 95% CI: 1.31–8.16) than those with *THY1*^low^ IGC tumors, with a median OS of 19 months compared to 69 months in the *THY1*^low^ group. In the GEO/Illumina cohort (Figure 6E), the same was observed (log-rank *p* = 0.0082, hazard ratio = 2.37, 95% CI: 1.22–4.58), with the *THY1*^high^ group showing a median OS of 32 months compared to 49 months in the *THY1*^low^ group. For the GEO/Affymetrix cohort (Figure 6F), the *THY1*^high^ group also showed a poor OS (log-rank *p* = 0.025, hazard ratio = 2.20, 95% CI: 1.09–4.46), with a median OS of 24 months compared to 64 months in the *THY1*^low^ group.

Regarding recurrence-free survival (RFS), although there are no data available for RFS of the GEO/Affymetrix cohort, in both the TCGA and GEO/Illumina cohorts, the *THY1*^high^ group showed a poor RFS. As depicted in Figure 6G, in the TCGA cohort, the *THY1*^high^ group showed poorer RFS (log-rank *p* = 0.018, hazard ratio = 3.58, 95% CI: 1.15–11.1), with a median RFS of 55 months compared to 64 months in the *THY1*^low^ group. In the GEO/Illumina cohort (Figure 6H), the same was observed (log-rank *p* = 0.0045, hazard ratio = 2.47, 95% CI: 1.30–4.69), with the *THY1*^high^ group showing a median RFS of 26 months compared to 84 months in the *THY1*^low^ group. Additionally, in the GEO/Illumina cohort, where adjuvant chemotherapy information was available, we verified whether *THY1* expression impacted the outcome of patients who used chemotherapy. As shown in Figure 6I, the patients with *THY1*^high^ IGC tumors who used the adjuvant chemotherapy regimen had a poor RFS (log-rank *p* = 0.05, hazard ratio = 2.62, 95% CI: 1.01–7.26), with a median RFS of 31 months compared to 84 months in patients with *THY1*^low^ IGC tumors who used the adjuvant chemotherapy. Additional information for these comparisons can be found in the Table S4. Moreover, information for all datasets from external GEO cohorts is available in Table S5. Altogether, these results highlight the impact of *THY1* expression on different aspects of patients’ prognosis.

## 4. Discussion

Through a feature selection method appropriate for gene expression data, SVM-RFE, and different classification approaches, supervised and unsupervised, we found that *THY1*^high^ IGC tumors have a distinct gene expression pattern compared to *THY1*^low^ tumors [40]. After validation in different external cohorts, a robust molecular signature of 35 genes (32 upregulated and 3 downregulated in the *THY1*^high^ group) demonstrated potential to discriminate between *THY1*^high^ and *THY1*^low^ IGC tumors, regardless of the transcriptome quantification methodology used (RNA-seq to TCGA and microarray to GEO) or geographic origin of the patients. This highlights its high potential for generalization and the consistency of the biological pattern found in this molecular signature. Given the geographic variation in gastric cancer incidence and that the microarray approach to transcriptome quantification represents approximately half of the transcriptome data available and is still widely used, it is important to find molecular patterns that are common regardless of these characteristics [58,59,60,61]. Therefore, we used this molecular signature to characterize a poor prognosis profile and to understand the underlying biology of these tumors [13]. Similarly, a previous work from Oh et al. (2018) reported a group of gastric tumors with a mesenchymal phenotype and poor prognosis characterized by differential expression of 299 genes, which included not only the *THY1* gene but also 9 genes present in our signature [62]. Furthermore, through modulation of *THY1* gene expression, Zhu et al. (2015) demonstrated that high expression of *THY1* leads to an inhibition of apoptosis and increased expression of *SPARC* in gastric tumor cells, with *SPARC* being found to be upregulated in the molecular signature identified in our work [35]. Moreover, Liu et al. (2021) showed that both *THY1* and *COL1A1* are potential hub genes in gastric adenocarcinoma associated with poor survival, with *COL1A1* being another member of the molecular signature identified in our work [16]. Although these studies did not address *THY1* directly and did not divide tumors by histological subtype, making comparison difficult, they corroborate our results, demonstrating that high expression of the *THY1* gene is accompanied by a distinct pattern of gene expression and that the group of 35 genes found as a molecular signature can successfully represent this pattern.

Analysis of the biological functions in which these molecular signature genes are involved showed that the epithelial–mesenchymal transition (EMT) is one of the most striking biological characteristics in *THY1*^high^ IGC tumors. This is an important cellular process in which epithelial cells adopt mesenchymal features [63]. In cancer, this process is widely described as having a central role in tumorigenesis by conferring tumor cells with migratory and invasive properties [64,65]. In agreement with our results, Shah et al. (2019) and Pajuelo-Lozano et al. (2020) showed that gastric cancer cells with high expression of *THY1* have characteristics of gastric cancer stem cells with an EMT phenotype and increased migration and invasion capacity [66,67]. Additionally, the processes of cell migration, motility, and locomotion were found to be enriched in the *THY1*^high^ group in the present work. Furthermore, the study by Oh et al. (2018), which demonstrated high expression of *THY1* in gastric tumors with a mesenchymal phenotype, seems to corroborate our results, though none of the previous studies directly addressed the IGC histological subgroup [62]. Nevertheless, together with our results, these previous data indicate that the EMT is a central biological process underlying the pathogenesis of *THY1*^high^ IGC tumors.

In addition to the EMT phenotype, several processes associated with remodeling of the tumor tissue composition and cell interaction with this tissue context were enriched, such as extracellular matrix organization, collagen formation, and angiogenesis. These processes can influence characteristics such as stiffness, density, and tissue perfusion, which are important factors in tumor progression [68,69,70]. Malanchi et al. (2011) found that cancer stem cells expressing *THY1* respond to increased matrix components at metastatic sites [71]. Moreover, Díaz Del Arco et al. (2022) showed that desmoplasia, a fibrotic process resulting from accumulation of extracellular matrix components, is associated with intestinal gastric cancer when compared to the diffuse type [72]. The Thy-1 protein, encoded by the *THY1* gene, is involved in mechano-signaling, a pathway that results from interaction with matrix components [73,74]. Thus, enrichment of these processes in *THY1*^high^ IGC tumors along with the EMT phenotype indicates a specific cellular and tissue context related to the pathogenesis of these tumors. Given that the molecular signature has been shown to represent a consistent biological pattern between different cohorts, our findings revealed the distinct molecular mechanisms by which the development and progression of *THY1* high tumors may occur.

When verifying the association between the *THY1*^high^ group and patient prognosis, we first found that the relationship between *THY1*^high^ tumors and clinicopathological staging is heterogeneous, with most evaluated cohorts demonstrating an association between *THY1*^high^ tumors and advanced stages. The fact that we observed *THY1*^high^ tumors in all stages, as well as *THY1*^low^, strengthens our hypothesis that *THY1*^high^ IGC tumors are a particular subtype and not a more advanced stage of the same tumor. Then, through survival analysis, we showed that patients with *THY1*^high^ IGC tumors have poorer OS than those with *THY1*^low^ IGC tumors. Furthermore, *THY1*^high^ IGC tumors also showed poor RFS. This confirms a previous result from our group that showed high *THY1* expression as a marker of poor OS in IGC patients, but now using multiple cohorts, we have been able to demonstrate that this is a consistent pattern [13]. These results agree with other previous studies, which found that the level of expression of the *THY1* gene in GC tumors was a marker of poor prognosis, though not directly in IGC [14,15,16]. Furthermore, we demonstrated the relationship between the *THY1*^high^ expression group and poor RFS among IGC patients, which had not been directly addressed by previous studies. These data indicate that patients with *THY1*^high^ IGC tumors are considerably more likely to experience early recurrence events than those with *THY1*^low^ IGC tumors, which is an important factor in the prognosis of gastric cancer and thus highlights the importance of *THY1* as a possible prognostic marker [4]. Interestingly, a poor RFS was also found for *THY1*^high^ patients when observing only those who used the adjuvant chemotherapy regimen. This fact may be related to the central role of EMT in *THY1*^high^ IGC tumors, as demonstrated by our previous results. The EMT process has already been shown to be an important factor in resistance to cancer therapy [75,76]. Additionally, chemo-resistant tumors have already shown high expression of *THY1* in other types of cancer, although there is no description in IGC [77]. In the study carried out by Oh et al. (2018), which addressed a mesenchymal profile of GC that has high *THY1* expression, a poor RFS in patients who used the adjuvant chemotherapy regimen was also observed [62]. This could indicate differences in the clinical benefit of adjuvant chemotherapy for this group of patients, and further studies are needed to address this question.

There are some limitations in our study, such as the bulk transcriptome approach. From these data, we cannot directly distinguish whether increased expression of the *THY1* gene in the tumor is a consequence of its increased expression in tumor cells, in mesenchymal cells of the tumor microenvironment, or both. Thus, further studies are needed to address the contribution of *THY1* expression in each of these populations to tumor biology. Nevertheless, this is one of the first studies to address the biological aspects of IGC tumors with high *THY1* expression, contributing to our understanding of the significance of this increase in gene expression and the concomitant molecular changes that occur in this context.

## 5. Conclusions

In this study, we identified a robust molecular signature of 35 genes for *THY1*^high^ IGC tumors, which highlights its distinct molecular pattern in relation to *THY1*^low^ tumors. This molecular signature was independent of possible methodological and population biases and was able to successfully discriminate between tumors with high and low expression of *THY1*. Our work sheds light on important processes underlying *THY1*^high^ tumor pathogenesis, such as the epithelial–mesenchymal transition and remodeling of the tumor tissue composition. Moreover, the *THY1*^high^ group showed a more advanced clinicopathological staging and a poor OS and RFS. Therefore, our data seem to indicate that *THY1*^high^ IGC tumors may be a particular subtype of gastric tumors that have a molecular profile that can explain the worse prognosis. We hope that these results will contribute to advancement of the current understanding of the *THY1* gene in IGC tumor biology and its potential use as a biomarker for the disease.

## Figures and Tables

**Table 1:** Thirty-five genes of the common molecular signature of *THY1*^high^ IGC tumors.

Gene Symbol	Description	L2FC	*p*-Value	Expression in *THY1*^high^
COL1A1	collagen type I α 1 chain	2.85	2.53 × 10^−49^	Upregulated
CCDC8	coiled-coil domain-containing 8	2.68	3.03 × 10^−31^	Upregulated
MFAP5	Microfibril-associated protein 5	2.51	2.78 × 10^−20^	Upregulated
TWIST2	twist family bHLH transcription factor 2	2.34	2.32 × 10^−31^	Upregulated
MXRA8	matrix remodeling-associated 8	2.31	6.94 × 10^−29^	Upregulated
CCL21	C-C motif chemokine ligand 21	2.28	2.48 × 10^−10^	Upregulated
CPXM1	carboxypeptidase X, M14 family member 1	2.25	3.74 × 10^−36^	Upregulated
TWIST1	twist family bHLH transcription factor 1	2.22	1.03 × 10^−21^	Upregulated
SPARC	secreted protein acidic and cysteine-rich	2.22	6.93 × 10^−60^	Upregulated
ANGPTL2	Angiopoietin-like 2	2.16	3.30 × 10^−29^	Upregulated
MFAP2	microfibril-associated protein 2	2.13	4.23 × 10^−17^	Upregulated
WNT2	Wnt family member 2	2.11	1.74 × 10^−13^	Upregulated
BIRC7	baculoviral IAP repeat-containing 7	2.10	6.36 × 10^−6^	Upregulated
COL6A2	collagen type VI α 2 chain	2.09	3.87 × 10^−40^	Upregulated
CTSK	cathepsin K	2.05	5.71 × 10^−34^	Upregulated
SERPINF1	serpin family F member 1	2.02	2.23 × 10^−27^	Upregulated
LYVE1	lymphatic vessel endothelial hyaluronan receptor 1	1.99	9.68 × 10^−12^	Upregulated
CD248	CD248 molecule	1.88	1.06 × 10^−38^	Upregulated
COL5A3	collagen type V α 3 chain	1.84	7.34 × 10^−21^	Upregulated
MEIS3	Meis homeobox 3	1.82	3.00 × 10^−34^	Upregulated
MSC	musculin	1.81	3.30 × 10^−29^	Upregulated
VSIG4	V-set and immunoglobulin domain-containing 4	1.80	5.93 × 10^−10^	Upregulated
DOK5	docking protein 5	1.75	1.56 × 10^−18^	Upregulated
MAGEE2	MAGE family member E2	1.72	9.09 × 10^−4^	Upregulated
LZTS1	leucine zipper tumor suppressor 1	1.70	9.92 × 10^−21^	Upregulated
MAFB	MAF bZIP transcription factor B	1.63	1.02 × 10^−17^	Upregulated
OSCAR	osteoclast-associated Ig-like receptor	1.60	2.33 × 10^−13^	Upregulated
PCDH19	protocadherin 19	1.60	1.71 × 10^−6^	Upregulated
HAMP	hepcidin antimicrobial peptide	1.54	2.17 × 10^−5^	Upregulated
COL15A1	collagen type XV α 1 chain	1.53	2.96 × 10^−17^	Upregulated
SLC11A1	solute carrier family 11 member 1	1.52	4.84 × 10^−14^	Upregulated
GPR26	G protein-coupled receptor 26	1.51	6.34 × 10^−3^	Upregulated
COL20A1	collagen type XX α 1 chain	−1.69	6.09 × 10^−5^	Downregulated
CXCL5	C-X-C motif chemokine ligand 5	−1.98	2.67 × 10^−3^	Downregulated
SPAG6	sperm-associated antigen 6	−2.44	5.66 × 10^−8^	Downregulated

In this table, the expression variable refers to the expression of a given gene in the *THY1*^high^ group compared to the *THY1*^low^ group.

**Table 2:** Gene Ontology terms and signaling pathways associated with the 35 molecular signature genes of *THY1*^high^ IGC tumors.

Overrepresentation Analysis	Signature Genes		*p*-Value	Database
	Up	Down		
Extracellular matrix organization	COL1A1, MFAP5, SPARC, MFAP2, COL6A2, CTSK, COL5A3, COL15A1	COL20A1	7.19 × 10^−9^	Reactome (R-HSA-1474244)
Collagen formation	COL1A1, COL6A2, COL5A3, COL15A1	COL20A1	1.17 × 10^−6^	Reactome (R-HSA-1474290)
Epithelial–mesenchymal transition	COL1A1, MFAP5, SPARC, COL5A3		2.77 × 10^−6^	Hallmarks
Positive regulation of cell migration	COL1A1, TWIST2, CCL21, TWIST1, SPARC, LYVE1		2.98 × 10^−4^	GOBP (GO:0030335)
Positive regulation of cell motility	COL1A1, TWIST2, CCL21, TWIST1, SPARC, LYVE1		3.74 × 10^−4^	GOBP (GO:2000147)
Positive regulation of locomotion	COL1A1, TWIST2, CCL21, TWIST1, SPARC, LYVE1		4.10 × 10^−4^	GOBP (GO:0040017)
Embryo development	COL1A1, MFAP5, TWIST1, MFAP2, MEIS3		2.18 × 10^−3^	GOBP (GO:0009790)
Epithelial–mesenchymal transition in colorectal cancer	TWIST2, TWIST1, SPARC		2.44 × 10^−3^	WikiPathways (WP4239)
Positive regulation of epithelial–mesenchymal transition	COL1A1, TWIST1		5.04 × 10^−3^	GOBP (GO:0010718)
Regulation of angiogenesis	TWIST1, SPARC, SERPINF1		1.22 × 10^−2^	GOBP (GO:0045765)

In this table, the up and down in the signature genes variable refer to the expression of a given gene in the *THY1*^high^ group compared to the *THY1*^low^ group.

## Data Availability

The raw data can be obtained from online databases, including the TCGA database (https://portal.gdc.cancer.gov/, accessed on 2 February 2023) under the study abbreviation TCGA-STAD and the GEO database (http://www.ncbi.nlm.nih.gov/geo, accessed on 19 February 2023) under the access numbers: GSE15459, GSE191275, GSE193453, GSE22377, GSE26899, GSE26901, GSE38749, and GSE57308.

## Supplementary Materials

The following supporting information can be downloaded at: https://www.mdpi.com/article/10.3390/genes15010028/s1, Table S1: Clinicopathological characteristics of patients from the TCGA cohort; Table S2: Differentially expressed genes between *THY1*^high^ and *THY1*^low^ IGC tumors from the TCGA cohort; Table S3: Data from external GEO cohorts used for validation; Table S4: Survival and clinicopathological association between *THY1*^high^ and *THY1*^low^ IGC tumors; Table S5: Characteristics and *THY1* classification of datasets from external GEO cohorts used for validation.
